# Efficient strategy for magnetic resonance image-guided adaptive radiotherapy of rectal cancer using a library of reference plans

**DOI:** 10.1016/j.phro.2025.100747

**Published:** 2025-03-03

**Authors:** Deqi Chen, Xiongtao Yang, Shirui Qin, Xiufen Li, Jianrong Dai, Yuan Tang, Kuo Men

**Affiliations:** a Department of Radiation Oncology, National Cancer Center/National Clinical Research Center for Cancer/Cancer Hospital, Chinese Academy of Medical Sciences and Peking Union Medical College, Beijing 100021, China; b Department of Oncology, Beijing Changping Hospital, Beijing 102202, China

**Keywords:** MRI-guided adaptive radiotherapy, Library of reference plans, Rectal cancer, MRI-Linac

## Abstract

**Background and purpose:**

Adaptive radiotherapy for patients with rectal cancer using a magnetic resonance–guided linear accelerator has limitations in managing bladder shape variations. Conventional couch shifts may result in missing the target while requiring a large margin. Conversely, fully adaptive strategy is time-consuming. Therefore, a more efficient strategy for online adaptive radiotherapy is required.

**Materials and methods:**

This retrospective study included 50 fractions from 10 patients with rectal cancer undergoing preoperative radiotherapy. The proposed method involved preparing a library of reference plans (LoRP) based on diverse bladder shapes. For each fraction, a plan from the LoRP was selected based on daily bladder filling. This plan was compared with those generated by conventional couch shift and fully adaptive strategies. The clinical acceptability of the plans (i.e., per protocol, variation-acceptable, or unacceptable) was assessed.

**Results:**

In per protocol criterion, 44 %, 6 %, and 100 % of the plans for LoRP, conventional couch shift, and fully adaptive strategies were achieved, respectively. In variation-acceptable criterion, 92 % of LoRP plans and 74 % of conventional couch shift plans were achieved. LoRP demonstrated 94 % target coverage (100 % prescription dose) in the fully adaptive strategy compared with 91 % in conventional couch shift strategy. The fully adaptive strategy had the best performance in sparing the intestine and colon. LoRP reduced the treatment session duration by more than a third (>20 min) compared with the fully adaptive strategy.

**Conclusion:**

LoRP achieved adequate target coverage with a short treatment session duration, potentially increasing treatment efficiency and improving patient comfort.

## Highlights

• Magnetic resonance-guided adaptive radiotherapy benefits from a plan library.
• Plans meeting criteria increased to 92%, up from 74% with the conventional method.
• Treatment time decreased from 55.1 to 34.9 min compared with fully adaptive method.

## 1 Introduction

Radiotherapy guided by magnetic resonance imaging (MRI) has recently been introduced for the treatment of rectal cancer [1], [2], [3], [4], [5], [6]. The superior soft tissue contrast and real-time imaging capabilities of MRI make it suitable for short-course radiotherapy of locally advanced rectal cancer (LARC) [7], [8], [9]. Conventional couch shift (including virtual couch shift) strategy, which corrects the target position at the prescribed isocenter, has limitations because it considers only rigid translations of anatomical structures and inadequately addresses bladder variations during the treatment course [10], [11]. Although the fully adaptive strategy considers anatomical changes in the target and organs at risk (OARs), the requisite delineation and full re-optimization extend the time interval between daily MRI acquisition and treatment delivery by an average of 15–29 min [12], [13]. The treatment session duration is important for radiotherapy accuracy and patient comfort [14]. Autosegmentation technologies can expedite the contouring process [15], [16], [17], [18], [19]. However, preparing a training dataset and generalizing the model for magnetic resonance (MR) sequences is challenging [20], [21], [22]. Moreover, even with the assistance of autosegmentation, time-consuming and resource-demanding manual adjustments are required [19], [23]. Thus, an easily implementable adaptive strategy that combines the efficiency of the couch shift strategy with the accuracy of the fully adaptive strategy is required.

The library-of-plans strategy involves preparing a set of plans from which one is selected based on the daily anatomy observed during pretreatment imaging. This strategy has been applied to address bladder filling variations for cone beam computed tomography (CBCT)-guided radiotherapy of the prostate [24], bladder [25], cervical [26], [27], [28], and rectal cancer [29], [30], [31], [32]. Boer et al. [33] first introduced the library-of-plans strategy to the MRI-Linac system; however, no dose-volume parameter evaluation was performed.

In this study, the library of reference plans (LoRP) strategy was applied to generate daily virtual couch shift plans for MRI-guided radiotherapy of rectal cancer. This approach was developed to obtain highly efficient and acceptable treatment plans that could save labor and reduce the duration of treatment sessions. To our knowledge, this is the first study to introduce the LoRP strategy into an MRI-guided radiotherapy platform. The dose-volume parameters were compared with those of conventional adaptive strategies.

## 2 Materials and methods

### 2.1 Patient data

The patient data comprised 50 fractions from 10 patients with LARC who underwent preoperative radiotherapy. All patients were randomly selected retrospectively from a phase III clinical trial (NCT05484024). Computed tomography (CT) scans were performed with patients having a full bladder. The patients were instructed to empty their rectum and bladder 1 h before the scan and then drink 1 L of fluid. The patients were treated in the supine position between November 2022 and April 2023 using the Unity system (Elekta AB, Stockholm, Sweden) [34], [35], [36]. Each patient received a prescribed dose of 25 Gy delivered in five fractions. Before each treatment fraction, a 3D T2-weighted MRI was acquired and registered to the simulation CT.

### 2.2 Region of interest and dose criteria

The target and OARs were delineated according to the National Cancer Center guidelines [37]. The gross tumor volume (GTV) included the rectal tumor as shown by colonoscopy and rectal MRI/pelvic CT scans as well as vascular invasion beyond the rectal wall. The gross tumor volume of the lymph nodes (GTV_nd_) included metastatic lymph nodes and cancer nodules in the rectal mesorectal, presacral, internal iliac, and obturator areas as shown by rectal MRI/pelvic CT scans. The clinical target volume (CTV) encompassed the primary lesion and extended 2 cm longitudinally along the rectal mucosa. It also included high-risk areas for lymphatic drainage and recurrence. An anterior margin of 1.5–2 cm was applied to account for bladder filling variations in the presacral, rectal mesorectal, and internal iliac lymphatic drainage areas. For patients with lymph node metastasis, the obturator lymphatic drainage area was also included. The planning target volume (PTV) encompassed an isotropic margin of 6 mm around the CTV. The simulation CT images were delineated prior to treatment planning. To obtain data for dose calculation and evaluation, the target and OARs (i.e., bladder, intestine, colon, femur, and perineum) were delineated retrospectively in the daily MRI.

### 2.3 Library of reference plan strategy

**Fig. 1:**
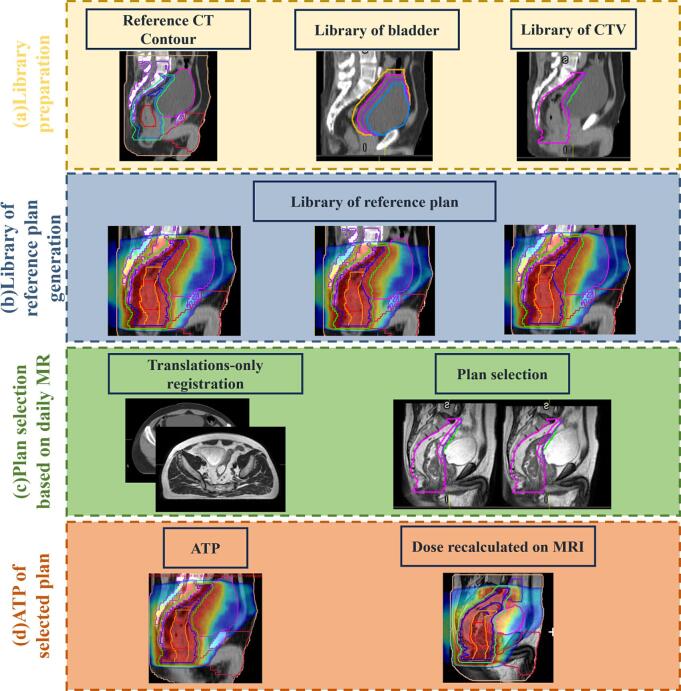
Workflow of the proposed method: (a) Preparation of bladder, clinical target volume (CTV), and planning target volume (PTV) libraries. (b) Preparation of the library of reference plans (LoRP). (c) Selection of a plan based on the daily magnetic resonance imaging (MRI). (d) Generation of the library of reference plans in adapt to position (LoRP-ATP) plan.

For the LoRP strategy, 7-field (215°, 260°, 325°, 35°, 100°, 145°, and 180°) intensity-modulated radiotherapy reference plans were generated on the simulation CT and optimized using the Monaco 5.40 treatment planning system (Elekta AB, Stockholm, Sweden). The LoRP was prepared using various bladders with variations in the anterior–posterior (AP) and superior–inferior (SI) directions (Fig. 1). The variation range was chosen based on the expected variation reported by Yee et al. [38] and Nijkamp et al. [11]. The LoRP strategy comprised the following four steps:

First, a library of bladders was prepared to account for shifts in the superior and posterior walls. Boolean operators (i.e., expansion or contraction) were used to create 16 bladder contours in the simulation CT, including the original. Shifts of 2 cm in the anterior direction to 1 cm in the posterior direction and 1 cm in the superior direction to 2 cm in the inferior direction were considered at 1-cm intervals (Fig. S1). A library of CTVs was created by subtracting the bladders in the library. An isotropic margin of 6 mm was applied to the CTV library to generate the PTV library.

Second, the LoRP was prepared by optimizing each reference plan with the same cost function for all OARs, where constraint optimization was applied to shrink margins to adapt to the updated structures. The target was replaced with one from the PTV library. The reference plans were optimized for fluence and segmentation.

Third, a plan was selected based on the daily MRI. Translation-only registration of the simulation CT scan was used to display the bladder contour on the daily MRI. The largest contour in the library of bladders that did not exceed the superior and posterior walls of the actual bladder was selected, ensuring that the selected CTV was minimized to protect OARs while remaining large enough to cover the target.

Fourth, Adapt-To-Position (ATP) using the “optimize weights and shapes from segments” approach was performed by adjusting fields for a virtual couch shift to align the target position with the selected LoRP (LoRP-ATP) plan on the daily MRI. The dose was calculated based on the simulation CT. To reflect the actual dose distribution, the LoRP-ATP plan was recalculated on daily MRI, where electron densities were assigned according to the contours.

### 2.4 Plan comparison and evaluation

Conventional ATP (cATP) and Adapt-To-Shape (ATS) treatment plans were generated and compared with the proposed LoRP-ATP plan. The cATP plan was generated with “optimize weights and shapes from segments” based on rigid registration with the daily MRI. The ATS plan was obtained by re-delineating contours on the daily MRI, followed by full re-optimization. Necessary adjustments were made by an experienced planner during the fluence optimization stage.

The LoRP-ATP and cATP plans for all 50 fractions were recalculated on the daily MRI, with electron densities assigned according to the contours to accurately reflect the actual doses delivered to the target and normal tissue. The ATS plans were optimized and calculated on the daily MRI, eliminating the need for further calculations. The performances of LoRP-ATP, cATP, and ATS were evaluated according to the clinical dose criteria (Table 1). The per protocol plan is considered the ideal plan. A plan is considered acceptable if it meets either the per protocol or variation-acceptable criterion. An unacceptable plan is one that does not meet either criterion.

**Table 1:** Dose constraints on target volume and normal tissue. A plan was considered acceptable if it achieved either the per protocol or variation-acceptable criterion. Per protocol reflected an ideal plan, and variation-acceptable reflected a near-ideal plan. A plan was unacceptable if the variation-acceptable criterion was not met.

Structure	Dose-volume parameter	Per protocol	Variation acceptable
PTV	V_25Gy_ [%] V_23.75Gy_ [%]	≥95 ≥99	≥90 ≥95
Intestine	V_27.5Gy_ [cm^3^]	<1	<5
Colon	V_27.5Gy_ [cm^3^]	<1	<5
Bladder	V_25Gy_ [%]	<50	<60
Femur L	V_25Gy_ [%]	<5	<10
Femur R	V_25Gy_ [%]	<5	<10
Perineum	V_20Gy_ [%] V_15Gy_ [%] V_10Gy_ [%]	<5 <35 <50	<10 <45 <65

The contouring and adaptive planning times were recorded to evaluate the efficiency of the different strategies. To consider their effects on the total treatment session duration, positioning, image acquisition, and delivery times were also recorded.

### 2.5 Statistics

Statistical analysis was performed using R (V4.2.3, R Foundation for Statistical Computing, Vienna, Austria). For each evaluated parameter, median and interquartile range (IQR) were recorded. The Shapiro–Wilk test was used to determine whether the data conformed to normal distribution. The paired *t*-test was used for normally distributed data. The Wilcoxon signed-rank test was used for non-normally distributed data. Differences between two sets of plans were considered statistically significant at p-values < 0.05.

## 3 Results

The bladder volume variations during treatment were obtained for all fractions along with the frequency of plans selected from the LoRP (Fig. S2). For most fractions, the bladder volume was smaller (50.5 % [IQR 37.8 %–70.3 %]) than that at the time of simulation. The most frequently selected plans had a 1 cm anterior shift and 2 cm inferior shift of the bladder (36 %), followed by a 1 cm anterior shift and 1 cm inferior shift (20 %) and a 2 cm shift both anteriorly and inferiorly (14 %).

**Fig. 2:**
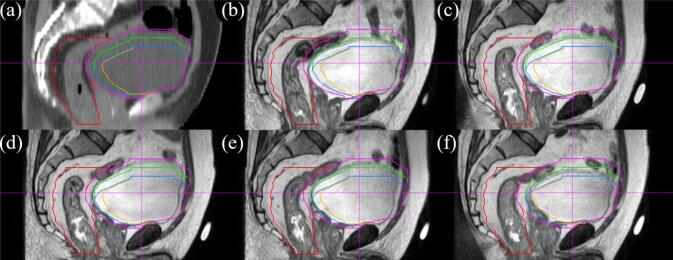
Simulation computed tomography (CT) scan and daily magnetic resonance imaging (MRI) of one patient: (a) Mesorectum in the simulation CT scan indicated by the red contour and bladder indicated by the pink contour. (b) Fraction 1: blue contour selected indicating a 1 cm anterior shift and 2 cm inferior shift. (c–e) Fractions 2–4: green contour selected indicating a 1 cm anterior shift and 1 cm inferior shift. (f) Fraction 5: orange contour selected indicating a 2 cm anterior shift and 2 cm inferior shift. The bladder shrank in all fractions compared to that in the simulation CT scan. (For interpretation of the references to colour in this figure legend, the reader is referred to the web version of this article.)

Fig. 2 illustrates the plan selection for one patient, showing the bladder contours from the simulation CT and the selected contours from the library in the daily MRIs of five fractions. The selection of bladder contours from the library was based on the shifts in the superior and posterior walls.

**Fig. 3:**
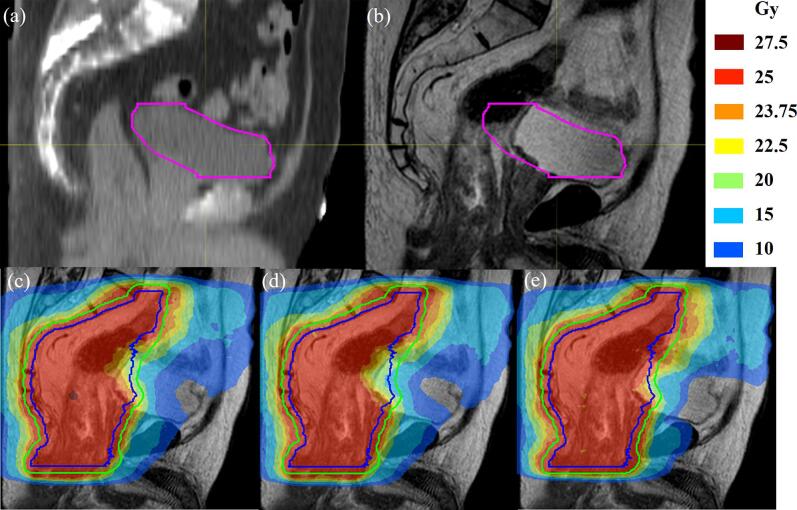
Fraction for which the library of reference plans in adapt to position (LoRP-ATP) and conventional adapt to position (cATP) plan underdosed the target: (a) Simulation computed tomography (CT) scan. (b) Magnetic resonance imaging (MRI) on the treatment day. (c) Dose distribution of LoRP-ATP. (d) Dose distribution of cATP. (e) Dose distribution of adapt to shape (ATS) plan. For this fraction, shrinkage of the bladder (pink) and gas filling of the colon and rectum caused a large offset of the anterior boundary of the target. LoRP-ATP, cATP, and ATS achieved target planning volume (PTV, green) coverages (100 % prescription dose) of 88.67 %, 87.76 %, and 95 %, respectively. The posterior wall of the bladder was 2 cm from the reference bladder, which was beyond the range of the library. In addition, gas in the colon pushed the target further forward. Although the LoRP-ATP plan achieved higher target coverage than cATP plan, which was insufficient to meet the dose criteria. (For interpretation of the references to colour in this figure legend, the reader is referred to the web version of this article.)

Approximately 44 % of the LoRP-ATP plans achieved per protocol, whereas only 6 % of the cATP plans achieved the criterion (Fig. S3). All ATS plans achieved per protocol. Meanwhile, 92 % of LoRP-ATP and 74 % of cATP plans achieved acceptable criterion, respectively. Fig. 3 shows an example of a LoRP-ATP plan that failed to satisfy the dose criteria, in which the gas in the rectum pushed the bladder wall beyond the range of the library.

**Fig. 4:**
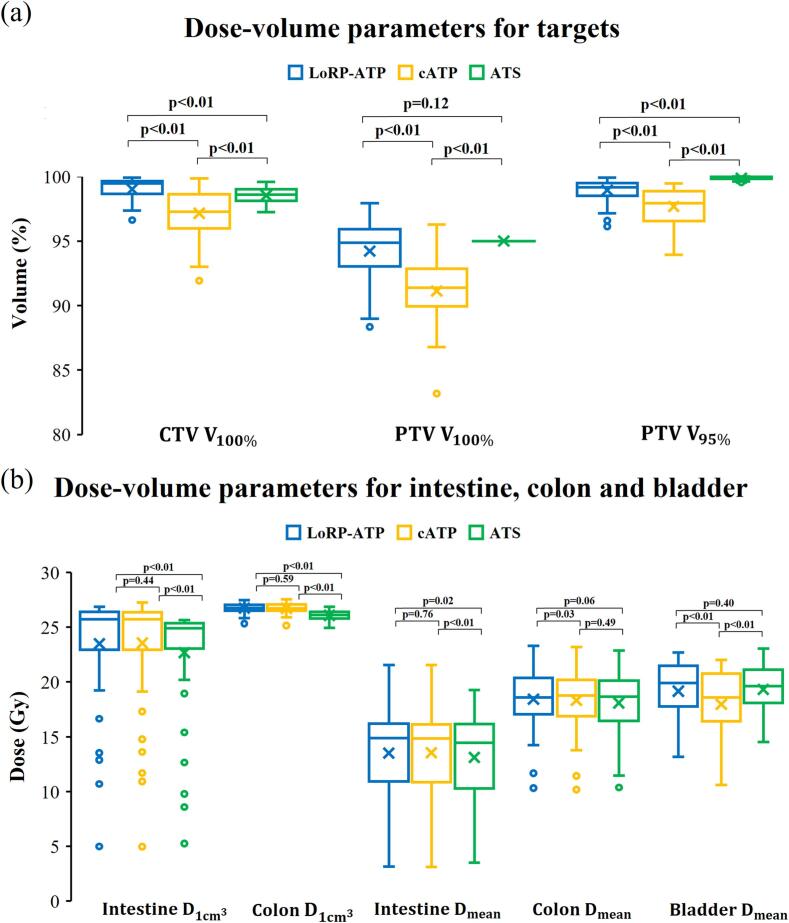
Box plots of dose-volume parameters: (a) the clinical target volume (CTV) coverage at 100 % prescription dose (25 Gy), the planning target volume (PTV) coverage at the prescription dose (25 Gy) and at 95 % of the prescription dose (23.5 Gy). (b) Near-maximum dose (1 cm^3^) of the intestine and colon, mean dose of the intestine, colon and bladder. The boxes represent the interquartile range (IQR), with the 25th (Q1) and 75th (Q3) percentiles. Horizontal lines within the boxes indicate the median values and X markers indicate the mean values. Outliers are displayed as individual data points and are defined as values below Q1 − 1.5*IQR or above Q3 + 1.5*IQR.

Fig. 4 shows a boxplot of the dose-volume parameters for each strategy. LoRP-ATP achieved the highest CTV coverage (100 % prescription dose). cATP achieved an average PTV coverage of 91 %, which increased to 94 % with LoRP-ATP and 95 % with ATS. There were no significant differences in target coverage between LoRP-ATP and ATS. LoRP-ATP achieved a similar bladder dose as ATS with no significant differences in the mean dose, V_25Gy_, V_20Gy_, and V_15Gy_. In contrast, cATP spared the bladder but reduced target coverage. LoRP-ATP and cATP showed comparable performance in protecting the intestine based on the metrics D_1__cm_^3^, D_mean_, V_25Gy_, and V_20 Gy_. Significant variations in the intestinal dose were observed among the different fractions, with minimal doses recorded when the intestine was far from the target area. However, potentially high doses were noted when the intestine overlapped with the target area, often due to inadequate bladder filling. ATS resulted in lower doses to the intestine and colon. However, when the bladder contracted, part of the colon fell within the irradiated area, leading to a slightly higher dose to the colon for LoRP-ATP than cATP based on V_25Gy_ (27.5 cm^3^ [IQR 17.4–51.1 cm^3^] vs. 23.1 cm^3^ [IQR 15.4–49 cm^3^], p < 0.05). However, LoRP-ATP and cATP showed no significant differences in D_1__cm_^3^, V_20Gy_, and V_15Gy_. All three strategies achieved similar performances for the femur and perineum (Table S1).

The contouring time was > 1h for the target and all OARs in the ATS strategy, particularly the intestine and colon. A practical ATS process required only 15.8 min for contouring the target and bladder and 11.3 min for the full plan re-optimization. In comparison, the optimization of ATP from segments required 6.4 min. All strategies had similar treatment session durations, including 2.8 min for positioning, 11.5 min for image acquisition and registration, 2.5 min for plan transfer and position verification, and 11.2 min for delivery. The plan selection for LoRP-ATP time was 0.5 min. Compared with ATS, LoRP-ATP decreased the treatment session duration by more than a third (55.1–34.9 min) but maintained acceptable plan quality for most fractions.

## 4 Discussion

The results of this study indicate that the proposed LoRP is a promising strategy for enhancing the efficiency and effectiveness of MRI-guided adaptive radiotherapy for rectal cancer. LoRP offers a balance between the rapid delivery of conventional couch shift and the comprehensive adaptability of fully adaptive strategies.

The primary advantage of the LoRP strategy is its ability to adapt to bladder filling variations without requiring the extensive time needed for full re-optimization, as is the case with ATS. This is particularly relevant given the frequent changes in bladder volume observed in this study. By creating a range of plans with different bladder volumes beforehand, the LoRP strategy allows the most appropriate plan to be selected quickly, reducing the treatment session duration while ensuring adequate target coverage. Although this study was based on the Unity system, the proposed method can be easily transferred to other MRI-guided radiotherapy platforms like the MRIdian (ViewRay, Cleveland, USA) [39], [40] and Aurora-RT systems (MagnetTx Oncology Solutions, Edmonton, Canada) [41], [42].

Library-of-plans strategies have been applied to CBCT-guided radiotherapy to address bladder inter-fraction variations in the prostate [24], bladder [25], and cervical cancer [26], [27], [28]. Previous studies have demonstrated the advantages of library-of-plans strategies for rectal cancer radiotherapy, showing reduced target volumes and improved coverage compared with conventional approaches [30], [31]. Beekman et al. [31] proposed an alternative approach using a population-based library with variable margins that consider rectum filling variations, potentially achieving further target volume reduction. However, large patient cohorts were required to establish a reliable statistical model, and a comprehensive dose-volume parameter evaluation was lacking. Conversely, this study incorporates anatomical variations in the anterior–posterior and superior–inferior directions, extending beyond the single-directional approach used by Lutkenhaus et al. [30]. The strategy with simple Boolean operators makes it robust and easy to implement, and the plan selection criteria are primarily based on bladder variations.

However, plan selection in CBCT-guided radiotherapy can be challenging because of limited image quality [31]. The superior soft tissue contrast of MRI facilitates accurate plan selection with clear organ boundaries. Furthermore, the MRI-Linac platform provides unique capabilities for real-time motion monitoring during treatment delivery without the need for contrast agents or implants [43]. Additionally, the integration of functional imaging can predict treatment outcome [44], [45]. These advantages make the implementation and evaluation of the library-of-plans strategy for the MRI-Linac system compelling. To our knowledge, this study represents the first dose-volume parameter evaluation of the library-of-plans strategy implemented on the MRI-Linac system. In addition, the dose calculations of the previous studies did not fully consider anatomical changes owing to the limited image quality and field of view of CBCT. Therefore, the dose calculations in the present study contribute to the advancement of research on the library-of-plans strategy.

The time saved by using LoRP compared with the ATS workflow involved two main components: contouring and optimization. Recent advances in autosegmentation for rectal cancer MRI have shown promise in reducing contouring time [15], [16], [17], [18], [19]. Although accurate contours are fundamental for achieving optimal target coverage and OAR sparing, current autosegmentation methods still require substantial manual adjustments, which limits their potential time savings [19]. Patient-specific autosegmentation has shown varying degrees of success across different anatomical sites, with reported time reductions ranging from 10 to 20 min for liver, kidney, and cervical cancer [46] to approximately 7 min for prostate cancer [47]. Despite these efforts, the autosegmentation model requires extensive training datasets. However, when LoRP’s library is prepared, LoRP-ATP plans can be easily integrated into the clinical workflow without additional manual adjustments. In terms of optimization efficiency, modifications to the ATS workflow can reduce processing time. The omission of fluence optimization might save 0.5–1 min per fraction; however, this approach risks compromising the plan quality.

It is important to acknowledge that library-of-plan approaches, regardless of methodology, face inherent limitations in addressing the full spectrum of anatomical variations. Compared with fully adaptive strategies, library-of-plan approaches achieve comparable target coverage but may result in higher doses for OARs [32]. In this study, LoRP-ATP provides a viable intermediate between cATP and ATS. For fractions in which the target and OARs on daily MRI are very similar to those in the simulation CT scan, cATP may be the most efficient strategy. For fractions with very large variations in the target (>2 cm shift), ATS may be most suitable. Between these two extreme cases, LoRP-ATP can improve the target coverage of cATP plans with higher efficiency than ATS. Adaptive strategy selection should consider treatment effectiveness, side effects, patient comfort, and bladder capacity.

One limitation of this study is that LoRP requires time for optimization. However, if the LoRP strategy is predefined, all reference plans can be generated automatically without additional human intervention because the library of targets was generated following the same rules and cost functions. In this study, the treatment plans can be optimized by the treatment planning system in the absence of other tasks. A library can be prepared in a single night and be used for all fractions. Additional manual approval may still require time and staff resources before treatment. Another limitation is the small sample size, which may limit the generalizability of the findings. This study focused on a specific subset of patients with rectal cancer, and the results may differ from those for other cancer types or patient populations. Future studies should focus on validating these findings using larger, more diverse patient cohorts and exploring potential refinements through population-based methodologies. Moreover, this was a retrospective study. Notwithstanding these limitations, the results indicate the high potential applicability of the LoRP strategy to MRI-Linac systems.

In conclusion, the proposed method achieved adequate target coverage with a short treatment session duration. Moreover, it improved plan acceptability compared with conventional couch shift adaptive plans and improved workflow efficiency compared with fully adaptive plans. However, the method was inferior to fully adaptive plans in terms of plan quality. Thus, the proposed method demonstrates the potential for enhanced treatment efficiency and patient comfort.

## Funding sources

This work was supported by the 10.13039/501100001809National Natural Science Foundation of China (12175312 and 12375320), CAMS Innovation Fund for Medical Sciences (2023-I2M-C&T-A-010), and the Summit Plan of Precise Tumor Radiation Treatment (2021-DF-003).

## CRediT authorship contribution statement

**Deqi Chen:** Investigation, Methodology, Writing – original draft. **Xiongtao Yang:** Data curation, Formal analysis. **Shirui Qin:** Software. **Xiufen Li:** Visualization. **Jianrong Dai:** Supervision. **Yuan Tang:** Project administration, Resources. **Kuo Men:** Conceptualization, Funding acquisition, Writing – review & editing.

## Declaration of competing interest

The authors declare that they have no known competing financial interests or personal relationships that could have appeared to influence the work reported in this paper.
