# Supplementary material for: Efficient strategy for magnetic resonance image-guided adaptive radiotherapy of rectal cancer using a library of reference plans
Source: Phys Imaging Radiat Oncol. 2025 Mar 3;33:100747. doi: 10.1016/j.phro.2025.100747 (PMC11926541; doi:10.1016/j.phro.2025.100747)
Supplement: Supplementary Data 1 [file mmc1.pdf]

Supplementary materials

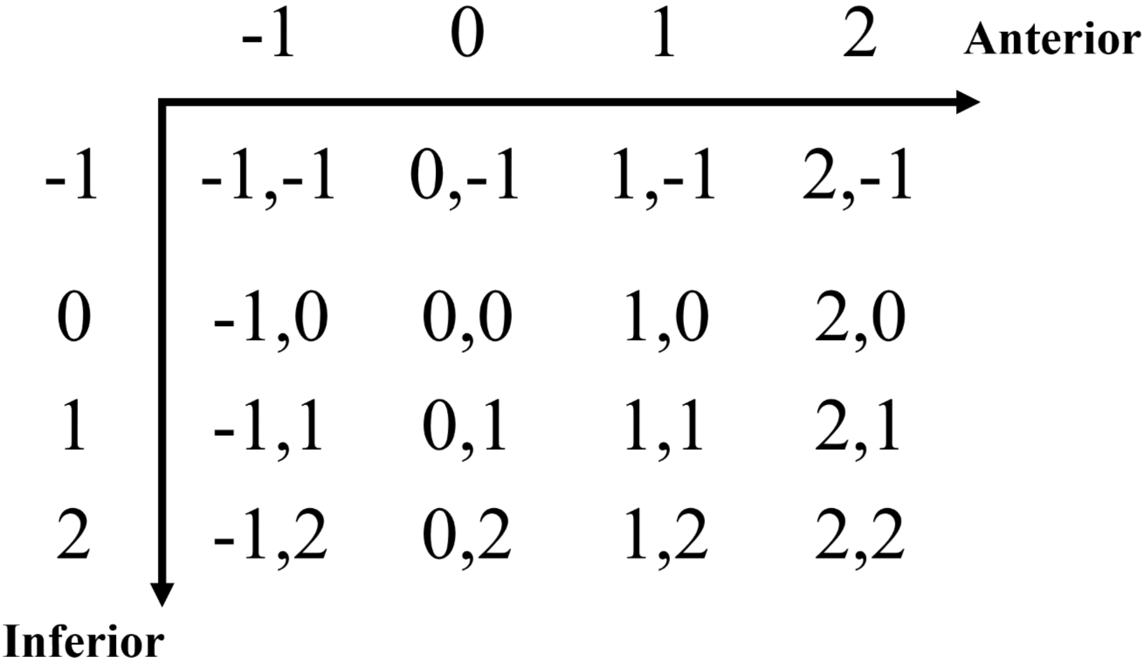

Supplementary Figure. S1. Preparation of library of bladders with variations in the posterior and superior boundaries. The X-axis indicates contraction (positive) or expansion (negative) of the posterior boundary. The Y-axis indicates contraction (positive) or expansion (negative) of the superior boundary. (0,0) denotes the contour in the simulation CT scan. In total, 16 contours including the original were created by Boolean operator (i.e., expansion or contraction) to the bladder contour in the simulation CT scan. The variations comprised ranges of 2 cm in the anterior direction to 1 cm in the posterior direction and 1 cm in the superior direction to 2 cm in the inferior direction in 1 cm intervals. Only the superior and posterior boundaries of the bladder were shifted, which affected the target position. Unit: cm.

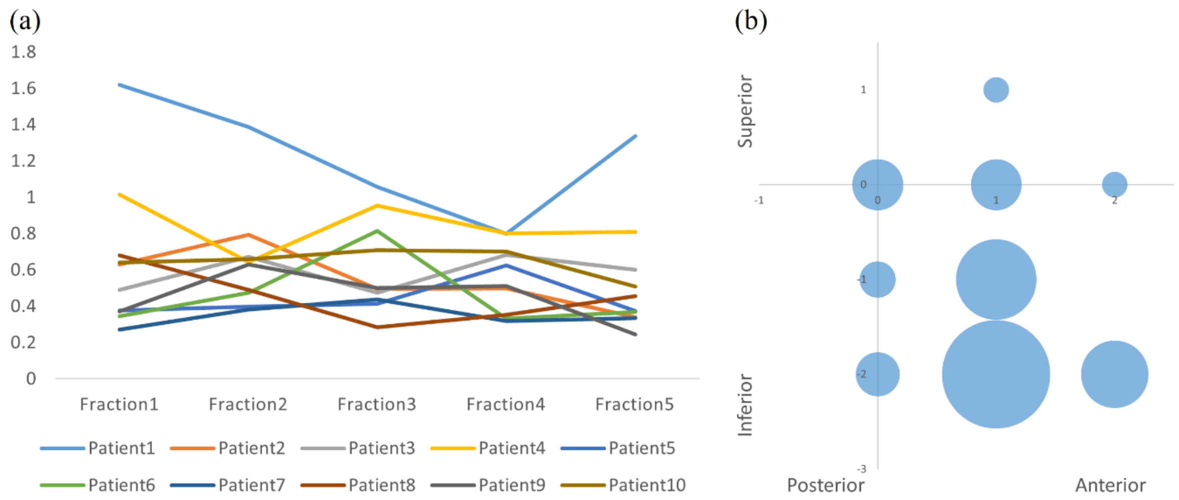

Supplementary Figure. S2. (a) Relative bladder volume. Volume in the simulation CT scan was normalized as 1. For most fractions, the bladder volume was smaller (60% in average) than that in the simulation, which could lead to underdosing of the target due to target movement caused by bladder shrinkage. The variation was irregular and unpredictable for the five fractions. (b) Frequency of LoRP selection. The horizontal line represents bladder variations in the AP direction. The vertical line represents bladder variations in the SI direction. Unit: cm. Three plans comprised 70% of the fractions: 1 cm anterior shift with 2 cm inferior shift, 1 cm anterior shift with 1 cm inferior shift, and 2 cm anterior shift with 2 cm inferior shift.

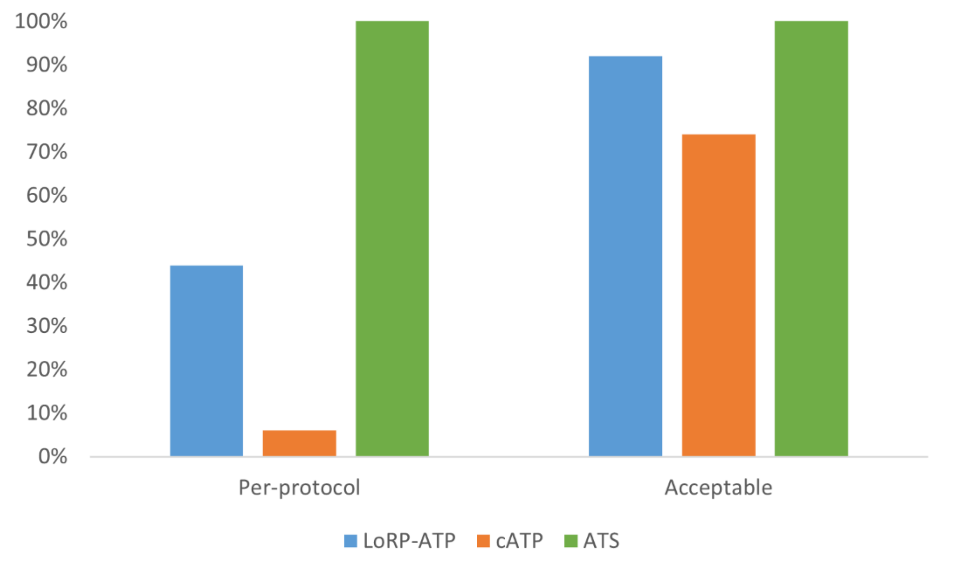

Supplementary Figure. S3. Plan acceptability rates of LoRP-ATP, cATP, and ATS.

Supplementary Table S1. Dose parameters. p-values: LoRP-ATP to cATP, cATP to ATS, and ATS to LoRP-ATP.

ROI	Parameter	LoRP-ATP	cATP	ATS	p-value between LoRP-ATP to cATP	p-value between cATP to ATS	p-value between LoRP-ATP to ATS
CTV	$V_{100\%}$ (%)	99.0±0.9	97.2±1.9	98.5±0.6	<0.01	<0.01	<0.01
	$V_{95\%}$ (%)	100±0.1	99.6±0.7	100±0	<0.01	<0.01	<0.01
PTV	$V_{100\%}$ (%)	94.2±2.4	91.1±2.6	95±0	<0.01	<0.01	0.12
	$V_{95\%}$ (%)	98.9±0.8	97.7±1.4	99.9±0.1	<0.01	<0.01	<0.01
	HI	1.1±0	1.2±0	1.1±0	<0.01	<0.01	<0.01
	CI	0.9±0	0.8±0	0.9±0	<0.01	<0.01	<0.01
Bladder	D_{mean} (Gy)	19.2±2.8	18.0±3.2	19.3±2.3	<0.01	<0.01	0.4
	V_{25Gy} (%)	17.4±7.6	6.9±7.1	16.1±5.8	<0.01	<0.01	0.22
	V_{20Gy} (%)	52.4±15.2	43.5±18.2	53.3±15.7	<0.01	<0.01	0.18
	V_{15Gy} (%)	74.7±19.8	71.0±21.8	75.6±17.2	<0.01	<0.01	0.22
Colon	D_{mean} (Gy)	18.4±2.7	18.3±2.7	18.1±2.9	0.03	0.49	0.06
	V_{25Gy} (%)	27.3±12.8	24.8±12.0	23.4±11.7	<0.01	0.10	<0.01
	V_{25Gy} (cm^3)	35.1±23.4	32.2±22.2	29.8±20.3	<0.01	0.03	<0.01
	V_{20Gy} (%)	59.8±35.7	60.0±36.4	59.2±36.0	0.80	0.83	0.52
	V_{15Gy} (%)	84.5±48.2	85.7±50.6	83.9±48.7	0.10	0.36	0.79
	D_{max} (Gy)	27.6±0.4	27.7±0.4	26.8±0.4	0.36	<0.01	<0.01
	D_{1cc} (Gy)	26.7±0.4	26.7±0.5	26.1±0.4	0.59	<0.01	<0.01
Intestine	D_{mean} (Gy)	13.5±3.9	13.5±4.0	13.1±4.1	0.76	<0.01	0.02
	V_{25Gy} (%)	5.3±5.9	4.7±5.1	1.8±2.4	0.18	<0.01	<0.01
	V_{25Gy} (cm^3)	10.3±12.7	9.0±11.1	3.7±5.5	0.20	<0.01	<0.01
	V_{20Gy} (%)	34.5±38.3	35.4±39.5	32.1±38.9	0.41	<0.01	0.04
	V_{15Gy} (%)	72.7±72.4	76.0±77.1	68.9±73.7	<0.01	0.03	0.19
	D_{max} (Gy)	25.3±4.2	25.3±4.2	24.1±4.3	0.72	<0.01	<0.01
	D_{1cc} (Gy)	23.5±4.8	23.5±4.8	22.7±5.0	0.44	<0.01	<0.01
Femur L	D_{mean} (Gy)	10.7±1.1	10.6±1.2	10.7±1.1	<0.01	0.31	0.77
	V_{25Gy} (%)	0.0±0	0.0±0	0.0±0	0.78	<0.01	0.03
Femur R	D_{mean} (Gy)	10.3±1.1	10.1±1.0	10.3±1.4	0.09	0.06	0.98
	V_{25Gy} (%)	0.0±0.1	0.1±0.4	0.0±0.0	0.55	0.57	0.65
Perineum	D_{mean} (Gy)	5.8±1.4	5.9±1.5	5.8±1.5	0.32	0.75	0.71
	V_{20Gy} (%)	0.8±1.1	0.8±1.1	0.9±1.1	0.97	0.20	0.22
	V_{15Gy} (%)	2.8±2.1	2.9±2.1	3.0±2.3	0.31	0.32	0.08
	V_{10Gy} (%)	16.2±7.1	16.9±7.8	16.9±8.6	0.08	0.43	0.05

Note: Conformity index (CI) was calculated according to Paddick's definition [1];

Homogeneity index (HI) was calculated as specified by the International Commission on Radiation Units and Measurements [2].

[1] Paddick I. A simple scoring ratio to index the conformity of radiosurgical treatment plans. Technical note. J Neurosurg 2000;93 Suppl 3:219–22. https://doi.org/10.3171/jns.2000.93.supplement.

[2] ICRU. Prescribing, recording, and reporting photon-beam intensity modulated radiation therapy (IMRT). ICRU Report 83. Journal of the International Commission on Radiation Units and Measurements 2010;10:106.
